# 5-Benzoyl-13-bromo-4-hy­droxy[2.2]paracyclo­phane

**DOI:** 10.1107/S1600536812013803

**Published:** 2012-04-13

**Authors:** Kelin Ma, Wenzeng Duan, Fuyan He, Yudao Ma

**Affiliations:** a School of Chemistry and Chemical Engineering, Shandong University, Jinan 250100, People’s Republic of China; b School of Chemistry and Chemical Engineering, Taishan University, Tai’an 27012, People’s Republic of China

## Abstract

The title compound, C_23_H_19_BrO_2_, was synthesized from 13-bromo-4-hy­droxy[2.2]paracyclo­phane and benzoyl chloride. The hy­droxy and carbonyl groups are involved in intra­molecular O—H⋯O hydrogen bonding. The crystal packing exhibits weak C—H⋯O inter­actions, which link the mol­ecules into sheets parallel to the *bc* plane.

## Related literature

For a related structure, see: Hong *et al.* (2011 ▶). For background to [2.2]paracyclo­phanes, see: Fache *et al.* (2000 ▶); Danilova *et al.* (2003 ▶). For details of the synthesis, see: Xin *et al.* (2010 ▶).
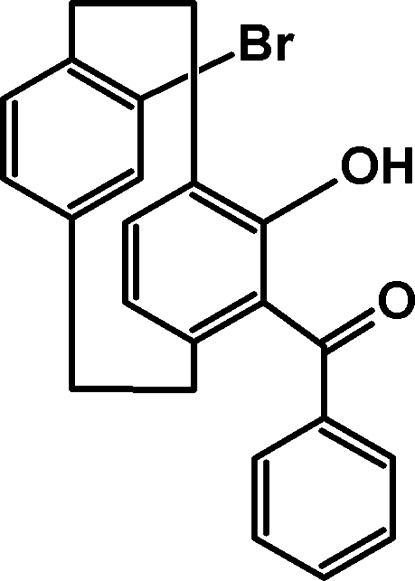


## Experimental

### 

#### Crystal data

C_23_H_19_BrO_2_
*M* *_r_* = 407.29
Monoclinic, 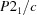

*a* = 12.5250 (18) Å
*b* = 7.8885 (12) Å
*c* = 19.143 (3) Å
β = 106.812 (3)°
*V* = 1810.5 (5) Å^3^
*Z* = 4
Mo *K*α radiation
μ = 2.29 mm^−1^
*T* = 273 K
0.10 × 0.10 × 0.08 mm

#### Data collection

Bruker APEXII CCD diffractometer
Absorption correction: numerical (*SADABS*; Bruker, 2007 ▶) *T*_min_ = 0.804, *T*_max_ = 0.838
7291 measured reflections
2586 independent reflections
1810 reflections with *I* > 2σ(*I*)
*R*_int_ = 0.033
θ_max_ = 23.3°

#### Refinement

*R*[*F* ^2^ > 2σ(*F* ^2^)] = 0.049
*wR*(*F* ^2^) = 0.147
*S* = 1.04
2586 reflections
236 parameters
H-atom parameters constrained
Δρ_max_ = 0.59 e Å^−3^
Δρ_min_ = −0.74 e Å^−3^

### 

Data collection: *APEX2* (Bruker, 2007 ▶); cell refinement: *SAINT* (Bruker, 2007 ▶); data reduction: *SAINT*; program(s) used to solve structure: *SHELXS97* (Sheldrick, 2008 ▶); program(s) used to refine structure: *SHELXL97* (Sheldrick, 2008 ▶); molecular graphics: *SHELXTL* (Sheldrick, 2008 ▶); software used to prepare material for publication: *SHELXL97*.

## Supplementary Material

Crystal structure: contains datablock(s) I, global. DOI: 10.1107/S1600536812013803/cv5263sup1.cif

Structure factors: contains datablock(s) I. DOI: 10.1107/S1600536812013803/cv5263Isup2.hkl

Additional supplementary materials:  crystallographic information; 3D view; checkCIF report

## Figures and Tables

**Table 1:** Hydrogen-bond geometry (Å, °)

*D*—H⋯*A*	*D*—H	H⋯*A*	*D*⋯*A*	*D*—H⋯*A*
O1—H1⋯O2	0.82	1.81	2.530 (5)	146
C4—H4⋯O2^i^	0.93	2.70	3.356 (7)	128
C19—H19⋯O1^ii^	0.93	2.69	3.404 (7)	134

Symmetry codes: (i) 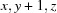; (ii) 
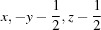
.

**Fig. 1.**
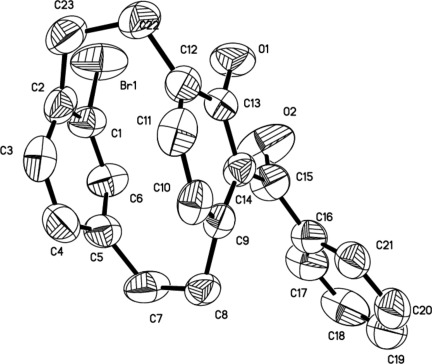
The molecular structure of (I) showing the atom numbering scheme and 50% probabilty displacement ellipsoids. The H atoms are omitted for clarity.

## supplementary crystallographic information

### Comment

The planar chiral Schiff bases have been used in many asymmetric reactions (Danilova *et al.*, 2003). [2. 2]Paracyclophane present planar chirality due to its configurationally rigid structure (Hong *et al.*, 2011). The salicyaldehyde derivative based on [2. 2]paracyclophane is the parent compound of various Schiff base ligands (Fache *et al.*, 2000). We reported here the crystal structure of the title compound (I), which is a derivative of [2.2]paracyclophane.

In (I) (Fig. 1), all bond lengths and angles are normal and in agreement with those observed in the related structure (Hong *et al.*, 2011). The mean planes A (C16-C21), B (C16/C15/O2), C (O2/C15/C14), D(C9-C14) and E (C1-C6) form the following dihedral angles: A/B 41.1 (2)°, C/D=18.4 (2)°, B/C=4.1 (2)° and D/E=1.6 (2)°. The hydroxy and carbonyl groups are involved in O—H···O hydrogen bonding (Table 1).

The crystal packing exhibits weak intermolecular C—H···O interactions (Table 1), which link the molecules into sheets parallel to *bc* palne.

### Experimental

The title compound was prepared by the method reported by Xin *et al.* (2010). The crystals were obtained by recrystallization from EtOH.

### Refinement

All the H atoms were located in difference maps, but placed in idealized positions (O—H 0.82 Å, C—H 0.93–0.97 Å), and refined as riding, with *U*_iso_(H) = 1.2-1.5 *U*_eq_ of the parent atom.

### Crystal data

**Table:**

C_23_H_19_BrO_2_	*F*(000) = 832
*M**_r_* = 407.29	*D*_x_ = 1.494 Mg m^−^^3^
Monoclinic, *P*2_1_/*c*	Mo *K*α radiation, λ = 0.71073 Å
Hall symbol: -P 2ybc	Cell parameters from 1805 reflections
*a* = 12.5250 (18) Å	θ = 2.4–20.5°
*b* = 7.8885 (12) Å	µ = 2.29 mm^−^^1^
*c* = 19.143 (3) Å	*T* = 273 K
β = 106.812 (3)°	Block, colourless
*V* = 1810.5 (5) Å^3^	0.10 × 0.10 × 0.08 mm
*Z* = 4	

### Data collection

**Table:**

Bruker APEXII CCD diffractometer	2586 independent reflections
Radiation source: fine-focus sealed tube	1810 reflections with *I* > 2σ(*I*)
Graphite monochromator	*R*_int_ = 0.033
phi and ω scans	θ_max_ = 23.3°, θ_min_ = 1.7°
Absorption correction: numerical (*SADABS*; Bruker, 2007)	*h* = −12→13
*T*_min_ = 0.804, *T*_max_ = 0.838	*k* = −7→8
7291 measured reflections	*l* = −21→21

### Refinement

**Table:**

Refinement on *F*^2^	Primary atom site location: structure-invariant direct methods
Least-squares matrix: full	Secondary atom site location: difference Fourier map
*R*[*F*^2^ > 2σ(*F*^2^)] = 0.049	Hydrogen site location: inferred from neighbouring sites
*wR*(*F*^2^) = 0.147	H-atom parameters constrained
*S* = 1.04	*w* = 1/[σ^2^(*F*_o_^2^) + (0.0807*P*)^2^ + 0.9145*P*] where *P* = (*F*_o_^2^ + 2*F*_c_^2^)/3
2586 reflections	(Δ/σ)_max_ = 0.006
236 parameters	Δρ_max_ = 0.59 e Å^−^^3^
0 restraints	Δρ_min_ = −0.74 e Å^−^^3^

### Special details

**Table:**

Geometry. All e.s.d.'s (except the e.s.d. in the dihedral angle between two l.s. planes) are estimated using the full covariance matrix. The cell e.s.d.'s are taken into account individually in the estimation of e.s.d.'s in distances, angles and torsion angles; correlations between e.s.d.'s in cell parameters are only used when they are defined by crystal symmetry. An approximate (isotropic) treatment of cell e.s.d.'s is used for estimating e.s.d.'s involving l.s. planes.
Refinement. Refinement of *F*^2^ against ALL reflections. The weighted *R*-factor *wR* and goodness of fit *S* are based on *F*^2^, conventional *R*-factors *R* are based on *F*, with *F* set to zero for negative *F*^2^. The threshold expression of *F*^2^ > σ(*F*^2^) is used only for calculating *R*-factors(gt) *etc*. and is not relevant to the choice of reflections for refinement. *R*-factors based on *F*^2^ are statistically about twice as large as those based on *F*, and *R*- factors based on ALL data will be even larger.

### Fractional atomic coordinates and isotropic or equivalent isotropic displacement parameters (Å^2^)

**Table:**

	*x*	*y*	*z*	*U*_iso_*/*U*_eq_	
Br1	0.94299 (6)	−0.03797 (10)	1.18603 (3)	0.1051 (4)	
C13	0.6602 (3)	0.0719 (6)	1.0485 (2)	0.0529 (11)	
C10	0.6311 (3)	0.3699 (6)	0.9720 (2)	0.0556 (11)	
H10	0.6135	0.4696	0.9451	0.067*	
O1	0.6717 (4)	−0.0722 (4)	1.08778 (19)	0.0844 (11)	
H1	0.6952	−0.1476	1.0667	0.127*	
C9	0.6706 (3)	0.2331 (5)	0.9423 (2)	0.0456 (10)	
C12	0.6440 (3)	0.2212 (6)	1.0841 (2)	0.0548 (11)	
C5	0.8972 (3)	0.2809 (7)	1.0081 (2)	0.0585 (12)	
C11	0.6170 (3)	0.3618 (6)	1.0413 (3)	0.0624 (13)	
H11	0.5882	0.4556	1.0591	0.075*	
C8	0.7383 (3)	0.2704 (5)	0.8900 (2)	0.0546 (11)	
H8A	0.7219	0.3845	0.8710	0.066*	
H8B	0.7164	0.1924	0.8491	0.066*	
C14	0.6714 (3)	0.0746 (5)	0.9773 (2)	0.0486 (10)	
C2	0.8655 (3)	0.3001 (7)	1.1481 (2)	0.0579 (12)	
C4	0.8811 (4)	0.4355 (7)	1.0375 (3)	0.0698 (14)	
H4	0.8816	0.5345	1.0113	0.084*	
C6	0.9229 (3)	0.1463 (7)	1.0567 (2)	0.0608 (12)	
H6	0.9494	0.0460	1.0423	0.073*	
C3	0.8642 (4)	0.4442 (7)	1.1062 (3)	0.0680 (14)	
H3	0.8517	0.5493	1.1244	0.082*	
C16	0.6713 (4)	−0.1147 (5)	0.8667 (2)	0.0587 (12)	
C15	0.6967 (4)	−0.0862 (6)	0.9465 (3)	0.0661 (13)	
O2	0.7342 (5)	−0.2069 (5)	0.9862 (2)	0.1206 (17)	
C7	0.8650 (4)	0.2539 (7)	0.9266 (2)	0.0744 (15)	
H7A	0.8892	0.1419	0.9167	0.089*	
H7B	0.9038	0.3363	0.9052	0.089*	
C17	0.7418 (5)	−0.2153 (6)	0.8396 (3)	0.0806 (15)	
H17	0.8073	−0.2593	0.8706	0.097*	
C20	0.5464 (5)	−0.0899 (8)	0.7459 (3)	0.0811 (16)	
H20	0.4804	−0.0479	0.7146	0.097*	
C21	0.5745 (4)	−0.0538 (6)	0.8193 (3)	0.0666 (14)	
H21	0.5271	0.0129	0.8372	0.080*	
C1	0.9105 (3)	0.1569 (6)	1.1249 (2)	0.0586 (12)	
C19	0.6139 (6)	−0.1852 (8)	0.7194 (3)	0.0922 (19)	
H19	0.5946	−0.2089	0.6697	0.111*	
C18	0.7114 (7)	−0.2484 (7)	0.7647 (4)	0.096 (2)	
H18	0.7577	−0.3139	0.7454	0.115*	
C22	0.6778 (4)	0.2271 (7)	1.1667 (2)	0.0710 (14)	
H22A	0.6724	0.1138	1.1851	0.085*	
H22B	0.6257	0.2987	1.1819	0.085*	
C23	0.7980 (4)	0.2950 (7)	1.2014 (2)	0.0724 (14)	
H23A	0.7938	0.4083	1.2201	0.087*	
H23B	0.8355	0.2231	1.2423	0.087*	

### Atomic displacement parameters (Å^2^)

**Table:**

	*U*^11^	*U*^22^	*U*^33^	*U*^12^	*U*^13^	*U*^23^
Br1	0.1093 (6)	0.1234 (6)	0.0873 (5)	0.0489 (4)	0.0358 (4)	0.0560 (4)
C13	0.055 (3)	0.053 (3)	0.051 (3)	−0.009 (2)	0.016 (2)	0.004 (2)
C10	0.052 (3)	0.046 (3)	0.059 (3)	0.008 (2)	0.000 (2)	0.000 (2)
O1	0.132 (3)	0.060 (2)	0.068 (2)	−0.011 (2)	0.040 (2)	0.0151 (18)
C9	0.050 (2)	0.038 (2)	0.043 (2)	0.0016 (18)	0.0045 (19)	−0.0004 (19)
C12	0.043 (2)	0.070 (3)	0.054 (3)	0.000 (2)	0.018 (2)	−0.008 (2)
C5	0.040 (2)	0.081 (4)	0.056 (3)	0.003 (2)	0.016 (2)	0.010 (3)
C11	0.051 (3)	0.061 (3)	0.071 (3)	0.015 (2)	0.011 (2)	−0.016 (3)
C8	0.074 (3)	0.043 (2)	0.043 (2)	−0.003 (2)	0.011 (2)	0.0063 (19)
C14	0.058 (3)	0.042 (3)	0.044 (2)	−0.0009 (19)	0.012 (2)	−0.0047 (19)
C2	0.046 (3)	0.074 (3)	0.047 (3)	−0.002 (2)	0.003 (2)	−0.007 (2)
C4	0.063 (3)	0.070 (4)	0.071 (3)	−0.015 (3)	0.011 (3)	0.014 (3)
C6	0.049 (3)	0.080 (4)	0.057 (3)	0.022 (2)	0.020 (2)	0.010 (3)
C3	0.061 (3)	0.065 (3)	0.070 (3)	−0.010 (2)	0.007 (3)	−0.018 (3)
C16	0.086 (3)	0.034 (2)	0.061 (3)	−0.008 (2)	0.029 (3)	−0.009 (2)
C15	0.098 (4)	0.033 (3)	0.067 (3)	0.004 (2)	0.024 (3)	0.002 (2)
O2	0.235 (5)	0.045 (2)	0.079 (3)	0.041 (3)	0.040 (3)	0.011 (2)
C7	0.068 (3)	0.107 (4)	0.055 (3)	0.014 (3)	0.028 (3)	0.018 (3)
C17	0.110 (4)	0.049 (3)	0.094 (4)	0.002 (3)	0.047 (3)	−0.007 (3)
C20	0.095 (4)	0.080 (4)	0.071 (4)	−0.032 (3)	0.027 (3)	−0.023 (3)
C21	0.078 (3)	0.063 (3)	0.065 (3)	−0.019 (3)	0.030 (3)	−0.017 (3)
C1	0.045 (2)	0.080 (4)	0.049 (3)	0.010 (2)	0.011 (2)	0.016 (2)
C19	0.133 (6)	0.076 (4)	0.076 (4)	−0.037 (4)	0.042 (4)	−0.024 (3)
C18	0.157 (6)	0.052 (4)	0.111 (5)	−0.018 (4)	0.092 (5)	−0.029 (3)
C22	0.069 (3)	0.094 (4)	0.057 (3)	0.000 (3)	0.029 (2)	−0.014 (3)
C23	0.070 (3)	0.098 (4)	0.050 (3)	−0.001 (3)	0.019 (2)	−0.014 (3)

### Geometric parameters (Å, º)

**Table:**

Br1—C1	1.903 (5)	C6—C1	1.362 (6)
C13—O1	1.348 (5)	C6—H6	0.9300
C13—C12	1.405 (6)	C3—H3	0.9300
C13—C14	1.411 (6)	C16—C21	1.374 (6)
C10—C9	1.376 (6)	C16—C17	1.394 (7)
C10—C11	1.390 (6)	C16—C15	1.484 (6)
C10—H10	0.9300	C15—O2	1.225 (5)
O1—H1	0.8200	C15—O2	1.225 (5)
C9—C14	1.418 (6)	O2—O2	0.000 (11)
C9—C8	1.516 (6)	C7—H7A	0.9700
C12—C11	1.362 (6)	C7—H7B	0.9700
C12—C22	1.513 (6)	C17—C18	1.397 (8)
C5—C4	1.383 (7)	C17—H17	0.9300
C5—C6	1.387 (6)	C20—C19	1.336 (8)
C5—C7	1.508 (6)	C20—C21	1.377 (6)
C11—H11	0.9300	C20—H20	0.9300
C8—C7	1.544 (6)	C21—H21	0.9300
C8—H8A	0.9700	C19—C18	1.370 (9)
C8—H8B	0.9700	C19—H19	0.9300
C14—C15	1.472 (6)	C18—H18	0.9300
C2—C3	1.389 (7)	C22—C23	1.554 (7)
C2—C1	1.391 (6)	C22—H22A	0.9700
C2—C23	1.503 (6)	C22—H22B	0.9700
C4—C3	1.392 (7)	C23—H23A	0.9700
C4—H4	0.9300	C23—H23B	0.9700
			
O1—C13—C12	116.4 (4)	O2—C15—O2	0.0 (4)
O1—C13—C14	121.8 (4)	O2—C15—C14	120.6 (4)
C12—C13—C14	121.7 (4)	O2—C15—C14	120.6 (4)
C9—C10—C11	121.2 (4)	O2—C15—C16	116.8 (4)
C9—C10—H10	119.4	O2—C15—C16	116.8 (4)
C11—C10—H10	119.4	C14—C15—C16	122.4 (4)
C13—O1—H1	109.5	O2—O2—C15	0 (10)
C10—C9—C14	116.8 (4)	C5—C7—C8	113.0 (4)
C10—C9—C8	117.2 (4)	C5—C7—H7A	109.0
C14—C9—C8	123.7 (4)	C8—C7—H7A	109.0
C11—C12—C13	115.8 (4)	C5—C7—H7B	109.0
C11—C12—C22	123.3 (4)	C8—C7—H7B	109.0
C13—C12—C22	119.7 (4)	H7A—C7—H7B	107.8
C4—C5—C6	115.7 (4)	C16—C17—C18	118.3 (6)
C4—C5—C7	121.3 (5)	C16—C17—H17	120.8
C6—C5—C7	121.8 (5)	C18—C17—H17	120.8
C12—C11—C10	122.3 (4)	C19—C20—C21	120.0 (6)
C12—C11—H11	118.8	C19—C20—H20	120.0
C10—C11—H11	118.8	C21—C20—H20	120.0
C9—C8—C7	112.4 (3)	C16—C21—C20	121.2 (5)
C9—C8—H8A	109.1	C16—C21—H21	119.4
C7—C8—H8A	109.1	C20—C21—H21	119.4
C9—C8—H8B	109.1	C6—C1—C2	121.7 (4)
C7—C8—H8B	109.1	C6—C1—Br1	118.4 (4)
H8A—C8—H8B	107.9	C2—C1—Br1	119.6 (3)
C13—C14—C9	118.8 (4)	C20—C19—C18	120.7 (6)
C13—C14—C15	117.9 (4)	C20—C19—H19	119.6
C9—C14—C15	122.9 (4)	C18—C19—H19	119.6
C3—C2—C1	114.8 (4)	C19—C18—C17	120.7 (6)
C3—C2—C23	120.0 (5)	C19—C18—H18	119.7
C1—C2—C23	123.7 (5)	C17—C18—H18	119.7
C5—C4—C3	120.6 (5)	C12—C22—C23	113.6 (4)
C5—C4—H4	119.7	C12—C22—H22A	108.8
C3—C4—H4	119.7	C23—C22—H22A	108.8
C1—C6—C5	121.8 (5)	C12—C22—H22B	108.8
C1—C6—H6	119.1	C23—C22—H22B	108.8
C5—C6—H6	119.1	H22A—C22—H22B	107.7
C2—C3—C4	121.7 (5)	C2—C23—C22	112.6 (4)
C2—C3—H3	119.2	C2—C23—H23A	109.1
C4—C3—H3	119.2	C22—C23—H23A	109.1
C21—C16—C17	119.1 (5)	C2—C23—H23B	109.1
C21—C16—C15	120.8 (4)	C22—C23—H23B	109.1
C17—C16—C15	120.0 (5)	H23A—C23—H23B	107.8

### Hydrogen-bond geometry (Å, º)

**Table:**

*D*—H···*A*	*D*—H	H···*A*	*D*···*A*	*D*—H···*A*
O1—H1···O2	0.82	1.81	2.530 (5)	146
C4—H4···O2^i^	0.93	2.70	3.356 (7)	128
C19—H19···O1^ii^	0.93	2.69	3.404 (7)	134

Symmetry codes: (i) *x*, *y*+1, *z*; (ii) *x*, −*y*−1/2, *z*−1/2.
